# Predictors of modern contraceptive use among reproductive age women in high fertility countries in sub-Saharan Africa: evidence from demographic and health surveys

**DOI:** 10.1186/s12905-022-02121-1

**Published:** 2022-12-13

**Authors:** Wubshet Debebe Negash, Habitu Birhan Eshetu, Desale Bihonegn Asmamaw

**Affiliations:** 1 Department of Health Systems and Policy, Institute of Public Health, College of Medicine and Health Sciences, University of Gondar, P.O. Box: 196, Gondar, Ethiopia; 2 Department of Health Education and Behavioral Sciences, Institute of Public Health, College of Medicine and Health Sciences, University of Gondar, Gondar, Ethiopia; 3 Department of Reproductive Health, Institute of Public Health, College of Medicine and Health Sciences, University of Gondar, Gondar, Ethiopia

**Keywords:** Predictors, Modern contraceptive, Reproductive age women, High fertility countries

## Abstract

**Background:**

The world’s population has increased faster than expected due to high fertility rates, with sub-Saharan Africa accounting for most of the increase. Modern contraceptive use is the best option to reduce the high fertility rate. There is limited information on the prevalence of modern contraception and its predictors in sub-Saharan Africa’s high-fertility countries. Therefore, this study aimed to assess the prevalence and predictors of modern contraception among reproductive-age women in high fertility countries in sub-Saharan Africa.

**Methods:**

We used Demographic and Health Survey data sets from the top 10 high fertility countries in sub-Saharan Africa. Stata version 16.0 software was used to analyze the data, and all statistical analyses were completed after the data had been weighted. Multilevel binary logistic regression was performed to identify factors associated with modern contraceptive use. Adjusted odds ratio with a 95% confidence interval, and a p value < 0.05 was used to declare statistical significance.

**Results:**

The prevalence of modern contraceptive use in all the countries considered in this study was 10.72% (95% CI 10.57, 10.87). In terms of the predictor variables, young aged women, those who had attended a primary or secondary level of formal education, women who received antenatal care follow up, women who reported distance to the health facility as not a big problem, and women from rich families were more likely to use modern contraceptives.

**Conclusion:**

Only one in 10 women of reproductive age used modern contraceptive methods in high fertility countries in sub-Saharan Africa. To improve the use of modern contraceptives, governments and non-governmental organizations studied in the countries should intensify programs that focus on those women who are economically poor, those with no formal education, no media exposure, and those with no antenatal care follow up.

## Background

The availability of various modern contraceptive methods empowered women to take control of their bodies, sexuality, and decisions about whether or not to have children [1, 2]. This, however, is dependent on their social, cultural, or economic status, as well as the country in which they live [1, 3]. Modern contraception is an important mechanism for controlling high fertility and improving the physical and economic wellbeing of women and their families [4–6].

In the developing world, modern contraception prevents an estimated 308 million unintended pregnancies each year [3]. Additionally, modern contraceptives decreases maternal and infant mortality [9] and reduce the risk of acquiring sexually transmitted diseases [7]. Apart from these benefits, it also contributes to a healthy and productive family, food security, and sustainable development in low and middle-income countries [8, 9].

Despite the aforementioned purposes, globally, in 2019, among the 1.9 billion women of reproductive age, only 842 million are using modern contraceptive methods, with sub Saharan Africa (SSA) having the lowest prevalence [10, 11] and about 47 million women have an unmet need for modern contraceptives [12].

Population growth and fertility decisions in SSA were potentially influencing the depletion of common resources, like land and/or water. It may occur when parents place a high value on having more children to help in housework or farmwork, or when parents are unaware of the benefits of fewer children [13, 14]. Lack of adequate supply or availability is one reason for contraceptive use lags in SSA. In addition, when FP services are available, there is often low demand and low utilization [15].

Another negative externality is that gender division of labour, gender norms, access to and control over resources and decision making dimensions have been intricately linked to the utilization of family planning [16]. The ability of women to seek health services and visit health care facilities depends on their decision making autonomy [17]. Similarly, in African contexts, studies indicate that restrictions on women’s autonomy by limiting their involvement in decision-making and financial independence results in lower utilization of healthcare services [17–19]. Multiple barriers, such as a lack of trust in Western medicine and a desire to have large families, as well as low socioeconomic status and access to family planning clinics, influenced contraception use [20].

Although different interventions like improvement in access and uptake, integration of family planning services in primary health care facilities, and education have been made to reduce fertility in SSA [21, 22], the world bank’s 2021 report on the global fertility rate lists ten SSA countries as having high fertility rates above 5 [23], which is higher than the worldwide and African average of 2.47 and 4.44, respectively [23]. Niger (6.82), Somalia (5.98), Democratic Republic of the Congo (5.82), Mali (5.79), Chad (5.65), Angola (5.44), Burundi (5.32), Nigeria (5.32), Gambia (5.15), and Burkina Faso (5.11) have the highest fertility rates [23].

Even though the prevalence of modern contraception has increased from time to time [24], its utilization in SSA is still challenged by a weak health system [25, 26]. The family planning 2020 goal is highly ambitious and has made less progress than expected [21, 27]. The prevalence of modern contraception use in SSA countries is different across the regions [4, 28–30]. Increasing the use of modern contraceptives in sub-Saharan Africa is a multi-faceted problem [31], that requires adequate and sustainable policies to increase the uptake of modern contraception in the region [32, 33]. This can help to improve future programmatic and policy directions [29].

Individual studies conducted in each country in Africa varied widely [7, 29, 34]. Despite recent study was tried to assess modern contraceptive in SSA [35], the study was restricted to adolescent girls and young women. All women of reproductive age (15–49 years) were included in this study, which makes it different from the previous study. Additionally, the former study was included 29 SSA countries. The current study, on the other hand, seeks to assess modern contraceptive utilization in the top ten high fertility SSA countries. Hence, it is essential to have a clear understanding of this issue in order to implement interventions that would curb population growth, improve the physical and economic wellbeing of women and their families.

Therefore, this study was intended to estimate the prevalence and factors associated with modern contraceptive use among reproductive-age women in high fertility sub-Saharan Africa countries using multilevel modelling based on the recent Demographic and Health surveys data. The findings of this study will eventually allow policymakers, program managers, non-governmental organizations, and other stakeholders involved in family planning to develop policies and programs for the regions based on the most recent evidence.

## Methods

### Study settings and data source

The study was a cross-sectional analysis of data from recent Demographic and Health Surveys (DHSs) conducted between January 2010 and December 2018 in nine countries in SSA. The DHS data are collected every 5 years in low-and middle-income countries using standardized, pre-tested, and validated questionnaires, and they follow a similar sampling, data collection, and coding procedure that allows for multi-country analysis. Niger, Democratic Republic Congo, Mali, Chad, Angola, Burundi, Nigeria, Gambia, and Burkina Faso were included in this study. These countries were selected because they are the top ten countries with high fertility rates in SSA, with fertility rates above 5.0, a higher value than the rate of 4.44 in SSA and 2.47 worldwide [23]. One country (Somalia) with no DHS data was excluded from the analysis. The data for these countries were obtained from the DHS program's official database, www.measuredhs.com, after permission was granted via an online request explaining the purpose of our study. We used the woman’s individual record (IR file) data set and extracted the outcome and explanatory variables. The DHS is a nationally representative household survey that uses face-to-face interviews on a wide range of population, health, nutrition tracking, and effect assessment measures. Respondents were selected using a two-stage stratified sampling technique. Enumeration Areas (EAs) were randomly selected in the first stage from the sampling frame (the frame are usually developed from the latest available national census), while households were selected by a systematic random sampling method from each cluster or EA in the second stage. Finally, interviews were conducted in selected households with target populations (women aged 15–49 and men aged 15–64). In this study, a total weighted sample of 159,014 married reproductive aged women who had given birth within the 5 years preceding the survey of each country were included. In addition, the reproductive aged women with missing values for the outcome variable were excluded from the study (Table 1).

**Table 1:** Description of Surveys and sample size characteristics in high fertility countries in SSA (n = 159,014)

Countries	Survey year	Weighted sample(n)	Weighted sample (%)
Angola	2015/16	14,379	9.04
Burkina Faso	2010	17,087	10.75
Burundi	2016/17	17,269	10.86
Chad	2014/15	17,719	11.14
DR Congo	2013/14	18,827	11.84
Gambia	2013	10,233	6.44
Mali	2018	10,519	6.62
Nigeria	2018	41,821	26.30
Niger	2012	11,160	7.02

### Study variables

#### Outcome variable

The outcome variable of this study is modern contraceptive use. Modern contraceptive use is defined as the current use of modern methods, including pill, intrauterine device, injections, male condom, female condom, female sterilization, implant/Norplant, and emergency contraception. The outcome variable was dichotomized as yes, if the women uses the above modern methods and coded as “1”, and those who were not using any of the aforementioned methods, those using traditional methods and/or folkloric methods were considered as not using modern methods and coded as “0”) [35–37].

#### Explanatory variables

This study used the following individual and community-level variables; the individual level variables includes; age, marital status, educational status, occupation, household wealth index, media exposure, ANC visit, number of living children, place of delivery and decision making for using contraceptive methods. Whereas, residence, distance to the health facility, and community level poverty were considered under the community level variables [35, 38, 39].

Community level poverty was generated by aggregating the individual level factors at cluster level and categorized it as high if the proportion is ≥ 50% and low if the proportion is < 50% based on the national median value since the data was not normally distributed [40].

#### Data management and statistical analysis

Stata version 16 software was used for data analysis. We began by calculating the modern contraceptive use among married women in nine sub-Saharan African countries with high fertility rates. Secondly, we appended the dataset and this generated a total sample of 159,014 women. After appending, we compute v005/1,000,000 (Women’s individual sample weight/1,000,000) to ensure weighted representative of the DHS sample and get reliable estimates and standard errors before data analysis.

For this study, four models were fitted: the null model, which had no explanatory variables, model I, which had individual-level factors, model II, which had community-level factors. And model III, which had both individual and community-level components. Since the models were nested, the Intra-class Correlation Coefficient (ICC), Median Odds Ratio (MOR) and Likelihood Ratio test (LLR), deviance (-2LLR) values were used for model comparison and fitness, respectively. Model III was the best-fitted model since it had the lowest deviance. Variables with a p value less than 0.2 in bivariable were used for multivariable analysis. Finally, in the multivariable analysis, adjusted odds ratios with 95% confidence intervals and a p value of less than 0.05 were utilized to identify predictors of modern contraceptive use.

**Ethical approval and consent to participate**

The study does not involve the collection of information from subjects. Consent to respondents is not applicable since the data set used in this study is freely available and possible to download from the link: https://dhsprogram.com/data/available-datasets.cfm. All the methods were conducted according to the Helsinki declarations. The data is available without respondents identifications. Approval was sought from MEASURE DHS/ICF International and permission was granted for this use.

## Results

### Prevalence of modern contraceptive use

**Fig. 1:**
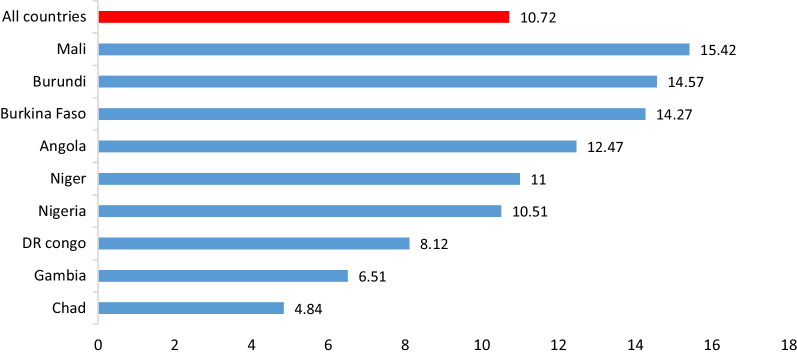
Prevalence of modern contraceptive use in selected high fertility countries in sub-Saharan Africa

Modern contraceptive use in all the countries was 10.72% (95% CI 10.57, 10.87), with Mali recording the highest prevalence of 15.42%, while Chad had the lowest prevalence of 4.84% (Fig. 1).

### Background characteristics of the respondents

A total of 159,014 weighted samples of reproductive age women in high fertility countries in SSA were included. Nearly sixty-four percent of women were from rural areas. Regarding education, 44.80% of the women had no formal education, while 22.13% had primary-level education. In terms of wealth status, 17.88% of the women fell in the poorest quintile, and 23.06% were in the richest categories (Table 2).

**Table 2:** socio-demographic and economic characteristics of respondents in high fertility countries (n = 159,014)

Variables	Categories	Frequency	Percent
Age in years	15–24	62,599	39.36
	25–34	51,707	32.52
	35 +	44,708	28.12
Place of residence	Urban	58,056	36.51
	Rural	100,958	63.49
Sex of household head	Male	127,349	80.09
	Female	31,665	19.91
Current marital status	Married	95,744	60.21
	Not married	63,270	39.79
Educational status of respondents	No education	71,232	44.80
	Primary education	35,185	22.13
	Secondary education	45,233	28.44
	Higher education	7362	4.63
Occupation of respondents	Not working	42,455	26.70
	Professional	51,132	32.16
	Agricultural	39,899	25.09
	Manual	17,678	11.12
	Others	7850	4.93
Wealth status	Poorest	28,433	17.88
	Poorer	30,204	18.99
	Middle	30,840	19.39
	Richer	32,872	20.67
	Richest	36,666	23.07
Community-level poverty	Low	84,375	53.06
	High	74,639	46.94
Having media exposure	Yes	100,938	63.48
	No	58,076	36.52
Visit the health facility for the last 12 months (n = 158,908)	No	82,177	51.71
	Yes	76,731	48.29
Distance to the health facility(n = 147,408)	Big problem	53,405	36.23
	Not big problem	94,003	63.77

### Random effect analysis

In the empty model, the values of Intra Class Correlation (ICC = 0.096) and Median Odds Ratio (MOR = 13.9) imply the presence of community-level variability of modern contraceptive use. Around 10% of the variation in modern contraceptive use is attributed to ICC. In the empty model, the presence of heterogeneity of modern contraceptive use between clusters is indicated by the MOR with a value of 13.9. It indicates that if we randomly select reproductive age women (15–49), a woman at the cluster with higher modern contraceptive use had around 13.9 times higher odds of modern contraceptive use than a woman at cluster with lower modern contraceptive use. Model III had the lowest deviance value (3501.5), and hence it was selected as the best-fitted model (Table 3).

**Table 3:** Model comparison and random effect analysis result in high fertility sun Saharan African countries (n = 159,014)

Random effect	Null model	Model1	Model 2	Model 3
ICC (%)	9.6	19.8	6.9	20.4
Variance	34.73	82.18	24.3	84.35
MOR	13.9	12.26	11.5	10.67
PCV	Reference	9.5	14	19.24
Deviance(-2Loglikelihood)	106,260.66	3532.64	100,262.64	3501.5

Intra class correlation cofficent

*MOR* median odds ratio, *PCV *proportional change in variance

### Obstetric characteristics of women

Nearly ninety percent of the women had antenatal care visits. Three-fourths (73.80%) of the respondents gave birth in the health facility. Regarding decision making for using contraceptive methods, 62% had joint decisions with husbands; the majority of the women (88.1%) knew about modern contraceptive methods (Table 4).

**Table 4:** Obstetrics-related characteristics of mothers in high fertility countries (n = 159,014)

Variables	Categories	Frequency	Percent
ANC visit	Yes	143,102	89.99
	No	15,912	10.01
Number of living children	1	63,421	39.88
	2–4	56,822	35.74
	5 +	38,771	24.38
Place of delivery	Home	41,664	26.20
	Health facility	117,350	73.80
Decision making for using contraceptive	Mainly respondents	4697	26.89
	Husband decision	1989	11.39
	Joint decision	10,782	61.72
Knowledge of modern contraceptive methods (n = 158,995)	Yes	140,211	88.19
	No	18,784	11.81

### Factors associated with the use of modern contraceptive methods

The odds of modern contraceptive use among women aged 15–24 and 25–34 years were 1.56 (95% CI 1.34–1.81) and 1.24 (95% CI 1.13–1.38) times higher than those aged 35–49 years, respectively. Women who had secondary/higher education and primary education had 3.30 (95% CI 2.92–3.73), and 1.36 (95% CI 1.22–1.52) times higher odds of modern contraceptive use, respectively as compared to those who did not attend formal education (no education). The category of women from households with the rich wealth index had 1.32 (95% CI 1.16–1.51) times higher odds of modern contraceptive use than those from poor households. The odds of modern contraceptive use was 1.68 (95% CI 1.51–1.86) times higher among women who had media exposure compared to no media exposure. The odds of modern contraceptive use was 1.33 (95% CI 1.1–1.6) times higher among those who had antenatal care compared to those who did not have antenatal care. The odds of modern contraceptive use was 1.17**(**95% CI 1.07–1.29) times higher among those women who reported distance to the health facility as not a big problem compared with their counterparts (Table 5).

**Table 5:** factors associated with modern contraceptive use among reproductive-age women in high fertility countries (n = 159,014)

Variables	Modern contraceptive use		Model 1 AOR (95% CI)	Model 2 AOR (95% CI	Model 3 AOR (95% CI)
	Yes n (%)	No n (%)			
*Individual level factors*					
Age in years					
15–24	4693 (7.5)	57,906 (92.5)	1.52 (1.31–1.77)		1.56 (1.34–1.81)*
25–34	7215 (13.95)	44,492 (86.05)	1.27 (1.15–1.40)		1.24 (1.13–1.38)*
35–49	5137 (11.49)	39,571 (88.51)	1		1
Educational status of the respondents					
No formal education	5403 (7.58)	65,831 (92.42)	1		1
Primary education	3727 (10.59)	31,458 (89.41)	1.36 (1.23–1.52)		1.36 (1.22–1.52)*
Secondary and higher	7914 (15.05)	44,681 (84.95)	3.21 (2.86–3.61)		3.30 (2.92–3.73)*
Wealth index					
Poor	3673 (6.26)	54,963 (93.74)	1		1
Middle	27.32 (8.86)	28,108 (91.14)	1.04 (0.91–1.18)		1.04 (0.91–1.19)
Rich	10,639 (15.3)	58,899 (84.7)	1.34 (1.19–1.52)		1.32 (1.16–1.51)*
Individual level media exposure					
Yes	13,489 (13.36)	87,450 (86.64)	1.67 (1.51–1.85)		1.68 (1.51–1.86)*
No	3555 (6.12)	54,520 (93.88)			1
Number of living children					
1	4627 (7.3)	58,794 (92.70)	1		1
2–4	7770 (13.67)	49,051 (86.33)	1.07 (0.94–1.22)		1.09 (0.95–1.25)
5 or more	4647 (11.99)	34,124 (88.01)	0.99 (0.84–1.17)		0.99 (0.84–1.17)
Antenatal care					
No	678 (4.26)	15,234 (95.74)	1		1
Yes	16,366 (11.44)	126,736 (88.56)	1.34 (1.12–1.62)		1.33 (1.1–1.6)*
Decision maker for contraception					
Jointly decision	8258 (76.59)	2523 (23.41)	1		1
Husband decision	1542 (77.53)	446 (22.47)	0.96 (0.84–1.20)		0.97 (0.85–1.10)
Mainly respondents	3680 (78.35)	1017 (21.65)	1.05 (0.95–1.15)		1.07 (0.97–1.17)
*Community level factors*					
Community level poverty					
Low	10,434 (12.37)	73,941 (87.63)		1	1
High	6610 (8.86)	68,029 (91.14)		0.73 (0.68–0.78)	1.16 (0.99–1.35)
Distance to the health facility					
Big problem	4673 (8.75)	48,732 (91.25)		1	1
Not Big problem	11,858 (12.61)	82,144 (87.39)		1.33 (1.28–1.38)	1.17 (1.07–1.29)*
Place of residence					
Rural	8305(8.23)	92,653 (91.77)		1	1
Urban	8739(15.05)	49,317 (84.95)		1.71 (1.64–1.78)	1.06 (0.94–1.20)

*Statistically significant at p value < 0.05

*AOR* adjusted odds ratio, *COR* crude odds ratio, Null model: adjusted for individual-level characteristics, Model 2: adjusted for community-level characteristics, Model 3: adjusted for both individual and community-level characteristics

## Discussion

This study aimed to investigate the prevalence and predictors of modern contraceptive use among reproductive-age women in high fertility countries in SSA. It was found that one in ten (10.72%) reproductive age women in these countries used modern contraception, with Chad having the lowest prevalence of 4.84%. The study findings revealed that age, level of education, wealth index, media exposure, ANC follow-up, and perceived distance to the health facilities were identified as the predictive factors of modern contraceptive use.

The prevalence of modern contraceptive use among reproductive-age women in the current study is lower than a study conducted in SSA [35]. The possible justification for the discrepancy might be the difference in study population and setting, in which the previous study was conducted in SSA among adolescent girls and young women but the current study is among all reproductive age women. As older age included in the study, the prevalence might be underestimated because getting older may make women believe they will not be able to become pregnant and, consequently, not use contraceptives. Additionally, the previous study was conducted in all SSA countries, whereas the current study is focused on the top ten high fertility countries. The study was also lower than studies conducted in Ethiopia [41–44], Nigeria [45, 46], Burundi [47], and Uganda [48]. The lower prevalence of modern contraceptive use in the current study compared with the previous studies could be explained by the difference in study settings, in which the current study used the top 10 high fertility SSA countries. In contrast, the previous studies were conducted at the individual countries. In addition, this discrepancy might also result from the socio-cultural differences between countries, which may have a dramatic effect on contraceptive use.

In this study, respondents aged 15–24 and 25–34 years old were more likely to use modern contraceptive methods than older age groups. It is consistent with the studies conducted in Ethiopia [49], Mali [50], Pakistan [51], and Bangladesh [52], which indicate that the proportion of women using modern contraceptive methods increases until it reaches its peak in the 30–34 age group. The possible explanation might be that women in the 15–24 and 25–34 age group is the time most women engaged in different activities such as attending school, income generation to fulfil their needs; as a result, they want to postpone their birth. Hence, to achieve their plan, they prefer to use contraceptives. On the other hand, getting older may make women believe they will not be able to become pregnant, which may lower the utilization of modern contraceptives [41, 53].

Another important factor that significantly influenced contraceptive use in this study was education. Women with primary and secondary/higher education were 1.36 and 3.30 times more likely to use modern contraceptives than women without formal education, respectively. The finding of this study is in agreement with those of studies conducted in low and middle-income countries [54, 55]. The possible reason might be women with formal education have better exposure to contraceptives through media and other ways of exposure, which improves access to contraceptive alternatives and helps them to understand the health benefits of the contraceptive in reducing fertility, unintended pregnancy, unsafe abortion, and other maternal and child problems [54, 56]. In addition, educated women have greater autonomy in decision-making regarding contraceptive use and need [56, 57]. These suggest that educating women will be one way to improve the use of modern contraception in these high fertility countries in SSA.

The likelihood of modern contraceptive use among women from households with rich wealth quintile was higher than those from households with poor wealth quintile. This finding is supported by studies done in developing countries [58]. The reason might be that women from rich households can be able to deal with the cost barrier associated with access to contraceptive use compared to those from poor households since they can overcome both the direct and indirect costs associated with contraceptive uptake [59]. Another possible reason could be that as an income increases, exposure to different information and financial accessibility of services will be improved. Which means that their income strongly influences their decision to visit the health facility, since the higher the socio economic status the better their health because of households’ increased ability to purchase health services [60, 61].

In this study, women who had media exposure were more likely to use modern contraceptives as compared to their counterparts. The finding is consistent with the evidence from other SSA countries like Ghana [62], Mali [63], and Nigeria [64], where exposure to mass media has a substantial positive effect on contraceptive use and intended future use of contraception. The reason for this might be that women exposed to media might have a better understanding of contraception, which can bring a positive change in their attitude toward contraception [64, 65]. As a result, considering mass media exposure as a predictor of modern contraceptive use, as well as using the media to raise awareness and share information, may be very important [66].

Another factor that predicts modern contraceptive use found in our study was ANC utilization; this is congruent with a population-based survey done in Kenya and Zambia [67], where women who had ANC follow up during pregnancy had higher odds of using modern contraceptives than their counterparts. This could be due to health care providers providing information during ANC follow up about family planning helps the mothers to agree and practice contraceptive use after delivery [68]. This suggests intervention on ANC attendant women is an effective strategy to increase modern contraceptive use.

Furthermore, the odds of modern contraceptive use among women who perceived distance to the health facility as not a big problem was higher than those women who perceived distance from the health facility as a big problem. This is in line with the studies conducted in Ethiopia [69] and Turkey [70]. The possible explanation could be that women who perceive distance as not a big problem are more likely to receive the recommended maternal health care services [71]. Furthermore, previous research has found that distance to health care facilities is a significant deterrent for women seeking health care [72, 73].

### Public health implications

Due to the high fertility rate, sub-Saharan Africa has contributed most of the world’s unexpected population growth. Modern contraception plays a crucial role in helping to regulate population growth, and to improve the physical and economic wellbeing of women and their families. However, in sub-Saharan African countries with high fertility, nine out of ten reproductive-age women did not use modern contraception. Thus, thousands of reproductive age women are not using any modern contraceptives. In turn, this can lead to an increase in unwanted or mistimed pregnancy rates, and sexually transmitted infections such as HIV/AIDS may be contracted as well. In order to combat the problem, nongovernmental organizations and policymakers should promote the use of modern contraception more widely in the region.

### Strengths and limitations

The main strength of this study was that it used nationally representative survey data and focused on high fertility countries in SSA. In addition, the DHS uses validated instruments in its appraisals of datasets along with the large sample size and well-designed procedures, such as training field enumerators and employing well-tested methods for data collection. However, since the surveys are cross-sectional, causality cannot be established for the findings. Moreover, the difference in the survey years may affect the comparability of the results since the modernization may have an impact on modern contraceptive utilization in more recent surveys compared to older ones. It is helpful to know what percentage of the “not married” women (which could involve women who are never married, divorced, separated, or cohabiting) are sexually active. However, such information is unavailable in the DHS data. Finally, it is important to note that some socio-cultural factors such as religion, which have direct or indirect effects on contraceptives use were not included.

## Conclusion

Only one in ten reproductive-age women used modern contraceptive methods in high fertility countries in sub-Saharan Africa, with Chad having the lowest prevalence. Age, educational status, wealth index, antenatal care follow-up, mass media exposure, and distance to the health facilities were the predictors for utilization of modern contraceptives. Therefore, governmental and non-governmental organizations should increase programs that improve these factors when designing interventions, especially in high fertility countries in SSA, to address the problem of low contraceptive use.

## Data Availability

Data for this study were sourced from Demographic and Health surveys (DHS) and available here: http://dhsprogram.com/data/available-datasets.cfm.

## Abbreviations

AOR: Adjusted odds ratio
CI: Confidence interval
DHS: Demographic and health survey
ICC: Intra-class correlation coefficient
MOR: Median odds ratio
PCV: Proportional change in variance
SD: Standard deviation
SSA: Sub-Saharan Africa
WHO: World Health Organization
